# Hypophysitis following Treatment with Ustekinumab: Radiological and Pathological Findings

**DOI:** 10.3389/fendo.2018.00083

**Published:** 2018-03-09

**Authors:** Ana M. Ramos-Leví, Manuel Gargallo, Ana Serrano-Somavilla, Miguel A. Sampedro-Núñez, Javier Fraga, Monica Marazuela

**Affiliations:** ^1^Department of Endocrinology, Hospital Universitario de la Princesa, Instituto de Investigación Princesa, Universidad Autónoma de Madrid, Madrid, Spain; ^2^Department of Endocrinology, Hospital Universitario Infanta Leonor, Madrid, Spain; ^3^Department Pathology, Hospital Universitario de la Princesa, Instituto de Investigación Princesa, Universidad Autónoma de Madrid, Madrid, Spain

**Keywords:** ustekinumab, hypophysitis, psoriasis, pituitary, autoimmunity

## Abstract

**Context:**

Ustekinumab is a human IgG1 monoclonal antibody that targets interleukin (IL)-12 and IL-23, which may be useful in the treatment of autoimmune conditions such as psoriasis, psoriatic arthritis, and Crohn’s disease. Hypophysitis is an immune-derived inflammatory condition of the pituitary gland that may lead to pituitary dysfunction. With the increasing use of immunotherapy, it is possible that this and other new immune-related adverse events (IRAEs) arise, although the mechanisms involved are still incompletely defined.

**Case description:**

A 35-year-old male, with a previous history of severe plaque-psoriasis who had started treatment with ustekinumab 4 months before, complained of progressive and persistent headache. Brain magnetic resonance imaging (MRI) was unremarkable. One year later, a new MRI was performed due to headache persistence, which revealed a homogenous and diffuse pituitary enlargement, with suprasellar extension and optic chiasm involvement, blurring of the pituitary stalk, absence of clear differentiation between the anterior and posterior lobes, and no signs of hemorrhage or adenomas. Endocrine evaluation was consistent with panhypopituitarism. Work-up of infiltrative and infectious diseases was negative. Follow-up MRI revealed an increase in the pituitary enlargement and transsphenoidal surgery was performed. Pathological findings revealed an intense fibrosis and a chronic inflammatory infiltrate, but no evidence of adenoma, granuloma, or acid fast bacilli. Immunohistochemical staining showed a combined T-cell (CD3+, CD4+) and B-cell (CD19+, CD20+) phenotype.

**Conclusion:**

We suggest a novel IRAE of ustekinumab, with full radiological and immunopathological iconography, which may be mediated by the complex interaction between different immunological processes.

## Background

Ustekinumab is a human IgG1 monoclonal antibody that targets interleukin (IL)-12 and IL-23. These two pro-inflammatory cytokines participate in immune functions, including the stimulation of natural killer cells and the differentiation of CD4+ T cells toward the T helper 1 phenotype (an action of IL-12), and the T helper 17 (Th17) pathway (an action of IL-23). Inhibiting their bioactivity may be useful in the treatment of autoimmune conditions such as psoriasis, psoriatic arthritis, and Crohn’s disease (1, 2).

Hypophysitis is an immune-derived inflammatory condition of the pituitary gland that may lead to pituitary dysfunction. With the increasing use of immunotherapy, it is possible that this and other new immune-related adverse events (IRAEs) will continue to arise.

We here describe the case of a patient who developed hypophysitis following ustekinumab treatment for psoriasis. We suggest potential mechanisms involved in its pathogenesis.

## Case Report

**Figure 1:**
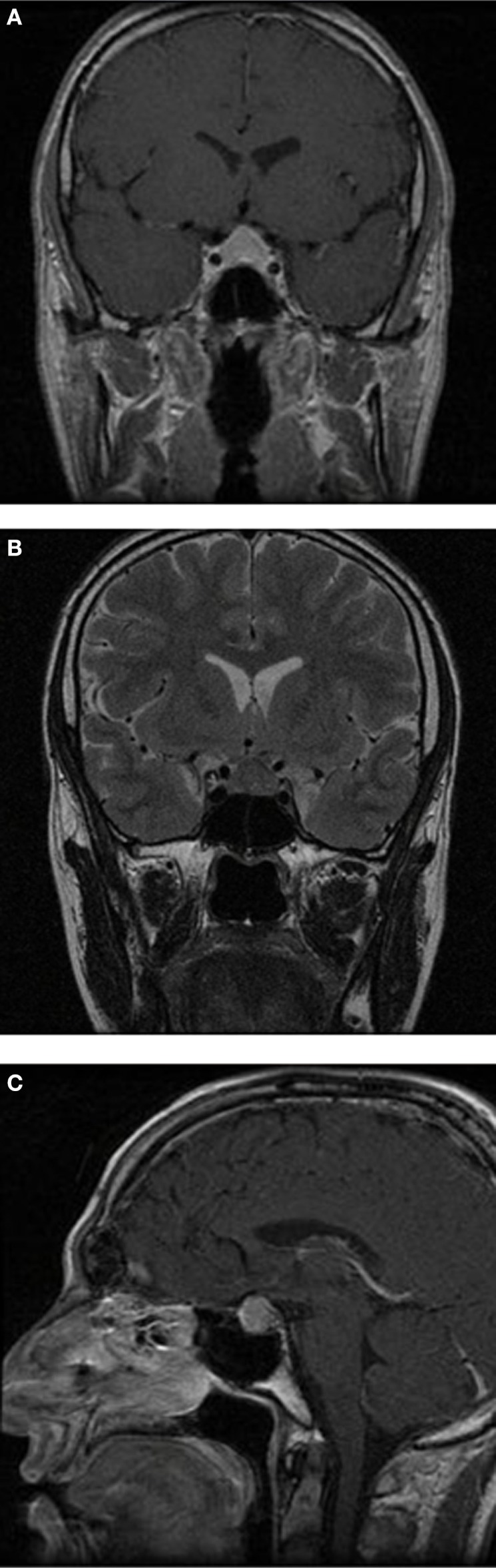
Brain magnetic resonance imaging (MRI) sections. Brain MRI, showing a homogenous and diffuse pituitary enlargement of up to 1.1 cm, which extended to the suprasellar region and contacted the optic chiasm, blurring of the pituitary stalk, absence of clear differentiation between the anterior and posterior lobes, and no signs of hemorrhage or adenomas. **(A)** Frontal T1-weighted after gadolinium; **(B)** frontal T2-weighted; and **(C)** sagittal T1-weighted after gadolinium.

A 35-year-old male, with a previous history of severe plaque-psoriasis who had started treatment with ustekinumab 4 months before, complained of progressive and persistent headache, which was refractory to non-steroidal anti-inflammatory drugs. Brain magnetic resonance imaging (MRI) was unremarkable. One year later, a new MRI was performed due to headache persistence, which revealed a homogenous and diffuse pituitary enlargement of up to 1.1 cm, which extended to the suprasellar region and contacted the optic quiasm, blurring of the pituitary stalk, absence of clear differentiation between the anterior and posterior lobes, and no signs of hemorrhage or adenomas (Figure 1). Visual fields were normal. The patient acknowledged absence of signs or symptoms suggestive of pituitary dysfunction, except for mild libido decrease and erection difficulties. Physical examination revealed multiple psoriatic plaques, with no other remarkable features. Laboratory work-up was consistent with hypogonadotropic hypogonadism, central hypothyroidism, central hypoadrenalism, and GH deficiency, but normality in the rest of the biochemical parameters evaluated (ions, proteins, lipid, renal, and hepatic profiles). Cerebrospinal fluid and work-up of infiltrative and infectious diseases, including sarcoidosis and tuberculosis, were negative. Follow-up MRI performed 6 months later revealed an increase in the pituitary enlargement to up to 1.7 cm. Because etiology of the mass was still unclear, the patient underwent transsphenoidal surgery of the sellar mass, with the purposes of both debulking and obtaining samples for biopsy.

**Figure 2:**
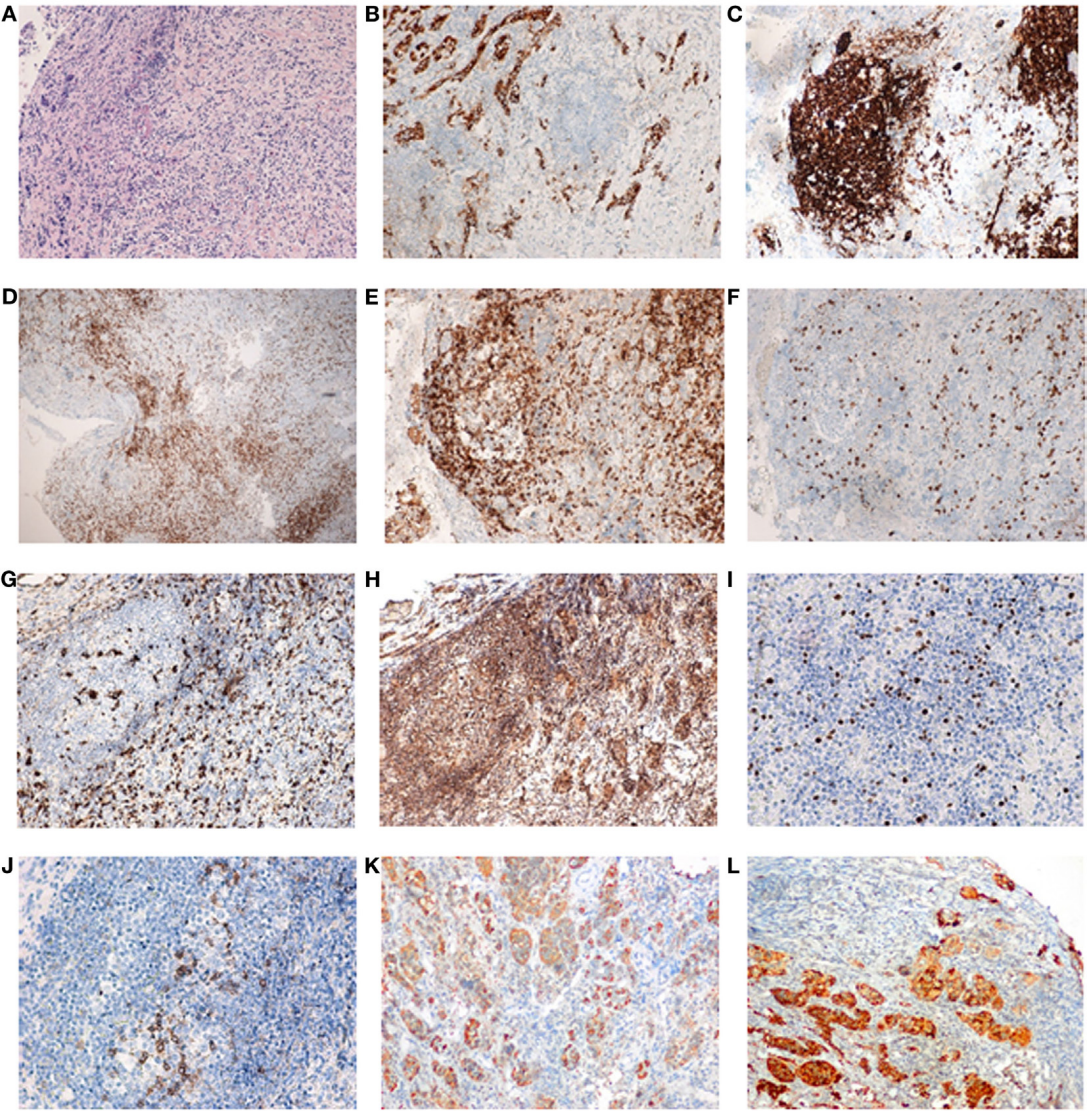
Pituitary gland serial sections from a representative area of the patient’s pituitary biopsy. Pituitary gland serial sections from a representative area of the patient’s pituitary biopsy, showing the most relevant immune-histopathological findings. Note the germinal center consisting of CD20+ B cells and peripheral CD3+ T cells (classified as T lymphocytes). Both CD4+ (helper) and CD8+ (cytotoxic) lymphocytes were found, as well as abundant macrophages (CD68+). T-regulatory cells were present in germinal centers and in the periphery (Foxp3+ and PD1+). Interleukin (IL)-17 staining was found not only in infiltrating lymphocytes but also in cells of pituitary origin in both hypophysitis and normal pituitary (data not shown). **(A)** Staining by hematoxylin–eosin; **(B)** synaptophysin; **(C)** CD20 (note how CD20+ cells form an aggregate, resembling a lymphoid follicle); **(D)** CD3; **(E)** CD4; **(F)** CD8; **(G)** CD68; **(H)** IL-17; **(I)** FOXp3; **(J)** PD1; **(K)** GH; and **(L)** prolactin. Sections **(A–H)** are shown as 10× and sections **(I–L)** as 20×.

Pathological findings revealed and intense fibrosis and a chronic inflammatory infiltrate of the pituitary gland with a high proportion of lymphocytes and some plasmatic cells, but no evidence of adenoma, granuloma, or acid fast bacilli. Immunohistochemistry pointed to a combined T-cell (CD3+, CD4+, CD8+) and B-cell (CD19+, CD20+) phenotype. Additional immune analysis showed positivity for FOXP3, PD1, and IL-17, and negativity for IgG4 and S-100 protein (Figure 2).

Ustekinumab was withdrawn, with subsequent improvement of headaches. Laboratory work-up after surgery revealed persistent panhypopituitarism, so hormonal replacement therapy was maintained. No other autoimmune disorders were evidenced in the clinical or analytical follow-up evaluation.

## Discussion

The use of ustekinumab for the treatment of psoriasis has progressively increased in recent years. Common side-effects include mild gastro-intestinal disturbance, dizziness, depression, cold symptoms, and artro-myalgias. More severe effects include allergic hyper-sensibility and opportunistic infections such as tuberculosis. However, although there is a potential risk of developing other IRAEs, this has been less explored (1).

Recently developed immunotherapeutic agents, which act as immune checkpoint inhibitors by releasing constraints on immune cells to promote antitumor activity, have generated promising results; specifically, the IgG1 monoclonal antibody Ipilimumab, which binds and inhibits cytotoxic T-lymphocyte antigen-4, or the IgG4 monoclonal antibodies Nivolumab and Pembrolizumab, which bind PD1. However, the immune-mediated antitumor effect frequently entails the development of other endocrine IRAEs, including hypophysitis, hypothyroidism, and adrenal insufficiency, especially in the case of Ipilimumab (3, 4). The reasons for these findings are still not fully understood but may concern modifications in the self-reactive effector T-cell repertoire and a further release of pituitary self-antigens, once pituitary inflammation and damage initiates, leading to the generation of more pituitary-reactive T-cells (4).

In this case report, hypophysitis possibly developed after treatment with ustekinumab, another monoclonal antibody. IgG4-mediated hypophysitis was reasonably ruled, given the negative pituitary IgG4 reactivity by immunohistochemistry, the absence of characteristic histopathologic findings, and the absence of any other clinical sign or symptom of IgG4-related disease at another site (5). Also, other causes of hypophysitis, such as histiocytosis, sarcoidosis, or tuberculosis, were reasonably ruled out with further clinical and laboratory work-up. It is true that a potential association between psoriasis and hypophysitis has been vaguely suggested (6), and lymphocytic and granulomatous hypophysitis have been anecdotally described in the setting of a past medical history of psoriasis, but with no clearly documented relationship (7, 8). However, in our patient, the absence of a correlation between the clinical course of both entities, the stability of skin plaques since the introduction of ustekinumab, the absence of any other autoimmune condition which could further be related to the potential development of hypophysitis, and the immunohistochemical pattern observed may reasonably exclude the role of psoriasis in the development of this patient’s hypophysitis.

We suggest for the first time a case of a possible ustekinumab-induced hypophysitis. In addition, apart from the biochemical work-up and radiological images, we provide novel immune-histopathological findings. We observed a predominance of T-cells (CD3+, CD4+), which is characteristic of lymphocytic hypophysitis (9), and no granulomas. Specifically, the positive phenotypic expression for FOXP3 and PD1 suggests the presence of T-regulatory cells (Tregs). Although finding Tregs in hypophysitis may seem paradoxical, it could be hypothesized that treatment with ustekinumab could originate aberrant Tregs and entail an imbalance in the lymphocytic profile (9).

On the other hand, IL-17 expression, which is characteristic of Th17 cells, was present in both infiltrating lymphocytes and also in pituitary cells in a similar grade as in normal pituitary tissue. Finding IL-17 expression in a similar grade in both infiltrating lymphocytes and normal pituitary cells could be due to the development of hypophysitis itself. In fact, T lymphocytes undergo activation in the pituitary gland and produce increased levels of ILs, including IL-17, perpetuating the alteration. Normal pituitary cells do not usually express IL-17, in contrast to infiltrating lymphocytes and pituitary cells in the setting of hypophysitis, which do so. Moreover, the plasticity of Th cells and the existence of at least two subsets of Th17 lymphocytes, pathogenic or pro-inflammatory, and non-pathogenic subsets, has been recently described (10) and can be identified by multiparametric flow cytometry, with analysis of the expression of the chemokine receptor CXCR3 (CD183), the P-glycoprotein 1 or multidrug resistance protein 1 (MDR1 or CD243), the differentiation molecule CD161, and the presence of IL-17 and IFN-γ. Moreover, IL-23 has been described to have a critical role on the differentiation of pathogenic Th17 cells (11). By contrast, non-pathogenic Th17 cells could participate in the regulation of the inflammatory phenomenon (12, 13). In this regard, it has been suggested that non-pathogenic Th17 cells can differentiate into Treg lymphocytes, contributing to the downregulation of autoimmunity and chronic inflammatory conditions (14). In view of these findings, we can suggest that if IL-23 levels are altered due to ustekinumab, the balance between Th17 pathogenic and non-pathogenic cells may also be altered, resulting, in an aberrant lymphocytic infiltration, meaning that hypophysitis may consequently develop and perpetuate. In this way, ustekinumab-induced hypophysitis could represent various the lymphocytic-mediated model.

In conclusion, we suggest a novel IRAE of ustekinumab, which may be mediated by the complex interaction between cytokines, Treg and different Th subsets, and we provide full radiological and immunopathological iconography. The presence of a previous history of psoriasis, which is an autoimmune disease, may disguise a full comprehension of the mechanisms involved, given the potential combination of concomitant immune alterations. Further tissue and peripheral blood evaluation, also in the setting of other immune checkpoint inhibitors, may help understand the broad spectrum of hypophysitis, and evaluate the possible different T-cell profile observed.

## Ethics Statement

The patient provided written informed consent regarding the procedures performed and publication of this case report.

## Author Contributions

AR-L contributed to study conception and design, followed-up the patient, researched, analyzed and interpreted data, and wrote the manuscript. MG followed-up the patient and researched and interpreted data. MS-N followed-up the patient. AS-S and JF performed the immuno-pathological analysis. MM contributed to study conception and design and reviewed and edited the manuscript. All authors contributed to the final version of this manuscript and approved it for publication.

## Conflict of Interest Statement

The authors declare that the research was conducted in the absence of any commercial or financial relationships that could be construed as a potential conflict of interest.
